# Advanced computer-aided detection system exhibits no more false positives than experienced endoscopists in an image-based comparative study of colon polyps

**DOI:** 10.3389/fmed.2026.1793448

**Published:** 2026-06-11

**Authors:** Shan Lei, Guanyu Zhou, Tyler M. Berzin, Xun Xiao, Peixi Liu, Pu Wang

**Affiliations:** 1 Department of Gastroenterology, Sichuan Provincial People’s Hospital, University of Electronic Science and Technology of China, Chengdu, China; 2 Center for Advanced Endoscopy, Beth Israel Deaconess Medical Center and Harvard Medical School, Boston, MA, United States

**Keywords:** artificial intelligence, colon polyp, colonoscopy, computer aided detection, false positive

## Abstract

**Background:**

The presence of computer-aided detection (CADe) false positives (FPs) may pose a hurdle to endoscopists’ confidence in, and acceptance of this technology. However, endoscopists also have FPs, which refer to the fact that the object they initially suspected was ultimately confirmed not to be a polyp. The objective of this study was to compare the FPs and overall diagnostic performance between CADe and experienced endoscopists through an image-based study.

**Methods:**

We prospectively enrolled 116 consecutive patients undergoing colonoscopy and diagnosed with pathologically confirmed small colon polyp, at Sichuan Provincial People’s Hospital. Images of polyps and polyp-free areas in an environment of colon inflation and deflation were taken. Randomly arranged images were labeled by CADe system and a group of experienced endoscopists. Sensitivity, specificity, and accuracy were compared between the CADe system and endoscopists.

**Results:**

The study comprised 464 images from 116 polyps and poly-free areas. The CADe system and endoscopists exhibited sensitivity, specificity, and accuracy of 90.09% vs. 87.56% (*P* = 0.46), 86.64% vs. 85.41% (*P* = 0.79), and 88.36% vs. 86.48% (*P* = 0.43), respectively, across all labeled images. In the test set with images taken in fully inflated lumen, sensitivity, specificity, and accuracy for the CADe system and endoscopists were 92.24% vs. 92.61% (*P* = 1.00), 95.69% vs. 86.70% (*P* = 0.04), and 93.97% vs. 89.66% (*P* = 0.13), respectively. In the test set with images taken in deflated lumen, sensitivity, specificity, and accuracy for the CADe system and endoscopists were 87.93% vs. 82.51% (*P* = 0.35), 77.59% vs. 84.11% (*P* = 0.24), and 82.76% vs. 83.31% (*P* = 1.00).

**Conclusion:**

The results indicate that a state-of-the-art CADe system exhibits a similar number of FPs as experienced endoscopists, while achieving high polyp sensitivity. Future study efforts to mitigate CADe false-positive distractions should focus not just on minimizing the false-positive rate, but also on educational, behavioral, or user-interface innovations to optimize CADe usability.

## Background and aims

Colorectal cancer (CRC) is a leading cause of cancer-related death globally (1). While colonoscopy remains the cornerstone for detecting and removing precancerous polyps (2), a notable proportion of polyps still evade detection routinely (3–9).

Challenges in polyp visualization, stemming from inadequate exposure, such as concealment behind folds or fecal matter, have gradually been mitigated through advancements in colonoscopy instrumentation, enhanced intestinal cleansing techniques, and standardized procedural training protocols (10, 11). Meanwhile, the failure to recognize visible lesions on the monitor, an equally important reason for missed polyps (12), is being addressed through cutting-edge artificial intelligence (AI) computer-aided detection (CADe) systems (13–23). Multiple CADe tools have been shown to augment screening and surveillance colonoscopy by increasing the adenoma detection rate (ADR), a key metric for the successful prevention of CRC (2, 24–26), however, the prevalence of false positives (FPs) may engender heightened procedural fatigue, extend examination durations and diminish user trust (27, 28). True CADe FPs, from a statistical perspective, are typically defined as any area that is tracked by the CADe alert box which is determined eventually not to be a polyp after inspection by the endoscopist. However, from the perspective of clinical practice, if a physician had diminished trust in a CADe tool, the endoscopists may succumb to “alert fatigue,” missing or ignoring meaningful CADe polyp alerts. Moreover, we have observed that even without using CADe, endoscopists routinely hone in areas of suspected polyps, for instance thickened folds, mucus or bubbles, which are eventually found to not to be polyps on closer observation. Practically speaking, these areas are dealt with in a fashion similar to CADe FPs. However, endoscopists may have different attitudes toward their own FPs vs. CADe’s FPs, with the latter feeling more obvious and potentially intrusive (29). Therefore, a clearer understand of how CADe FPs compare to endoscopist FPs may support a more nuanced understanding of how both types of FPs may influence the performance of the “physician-AI” hybrid during screening colonoscopy.

The aim of this study is to undertake a direct comparative analysis of FPs between experienced endoscopists and a state-of-the-art CADe system, on a wide variety of prospectively-collected colonoscopy images, and analyze whether a certain degree of FPs will lead to a higher detection rate.

## Materials and methods

### Study design and participant enrollment

This study was designed to perform a direct head-to-head comparison between CADe systems and endoscopists under uniform input information and observation conditions, and to eliminate rule-induced bias introduced by the predefined rule specifying the number of consecutive positive frames required to trigger an on-screen alert box. Consecutive patients with histologically confirmed small colorectal polyps who underwent colonoscopy at the Endoscopy Center of Sichuan Provincial People’s Hospital, China, between March 2020 and August 2020, were prospectively enrolled in this study. We collected four high-definition images on each enrolled patient by using the following protocol: initial images of each polyp were captured with the colon lumen fully inflated, followed by intentional deflation to induce lumen wrinkling without moving the lens, ensuring the visibility of the polyp, after which the images were retaken. All images were taken at a distance of at least 3 cm, to simulate challenging moments of initial polyp recognition at a distance. The same methodology was applied to well-inflated and underinflated lumens in polyp-free areas within the same segment of the colon.

All high-definition images were captured at a resolution of 1,920 × 1,080 pixels using processors from Olympus EVIS LUCERA CV290 (SL) (Olympus Medical Systems Co., Tokyo, Japan), Fujinon 4450 HD (Fujinon Toshiba ES Systems Co., Ltd., Tokyo, Japan), and Sonoscope HD-550 (SonoScope Bio-medical Technology Co., Ltd., Shen Zhen, China), along with adapted high-definition colonoscopes. This study received approval from the Ethics Review Board of Sichuan Provincial People’s Hospital. All patients have signed informed consent forms. All data used in this study was de-identified for confidentiality purposes.

### Schedule

An independent research assistant used computer random shuffling method to arrange the images in an unordered manner in the folder. We used a previously validated deep learning CADe system (EndoScreener, Shanghai Wision AI Co., Ltd., China) to screen and label the polyps in images with bounding boxes, with an operating point predefined as 0.3 to balance sensitivity and specificity (17). This CADe system is a real-time automatic polyp detection system, which was developed on a deep learning architecture, it was trained and fully validated to have over 95% sensitivity and specificity to detect polyps (17) before the initiation of this study. The subsequent upgraded versions of this system, with basic design architecture unchanged, have achieved improved sensitivity, specificity, and accuracy, and has been verified in multiple clinical studies to significantly improve the adenoma detection rate (ADR) and reduce the adenoma miss rate (AMR) during colonoscopy (3, 12, 18, 20, 29).

Seven experienced endoscopists, who have over 10 years of independent colonoscopy and polypectomy experience, with a completion of over 5,000 cases, participated in the evaluation of the images. These endoscopists were not directly involved in the design of the study, the colonoscopy procedures, or the randomization of images. The endoscopists were asked to review the images and label any areas they suspect to be polyps on the provided still images. Each image was only allowed to be viewed once within 60 s and could not be reviewed twice or modified. In order to collect data on physician thought process and certainty level, they were required to use red boxes to label polyps where there was high-certainty, and to use blue boxes to label possible polyps that were of moderate or low certainty based on the provided image. All labeled images were finally statistically analyzed by the research assistant based on the correct results.

### Outcomes and definitions

When a labeled lesion is confirmed as an actual polyp, then it is considered a true positive (TP). The absence of the label on an actual polyp is counted as a false negative (FN). Thus, the total number of TPs and FNs is equal to the total number of polyps presented in the images. Therefore, sensitivity equivalent to TP rate (TPR) was defined as TP divided by total number of polyp appearances = TP/(TP + FN). If there is no label on an image without polyps, the image is counted as a true negative (TN). A FP is defined as any label in a non-polyp area. FP rate (FPR) was defined as the ratio of false positives to the total number of non-polyp images, calculated as FPR = FP / (FP + TN). Therefore, specificity was defined as TN divided by total number of images without polyp = TN/total number of images without polyp. This is the commonly accepted statistical method for evaluating image detection. Accuracy was defined as TP plus TN divided by total number of images = (TP + TN)/ total number of images.

We analyzed the performance of CADe and endoscopists to detect polyps in cases of fully inflated lumen and underinflated lumen, respectively, attempting to analyze how CADe and humans endoscopists perform in optimal (inflated) vs. non-optimal (underinflated) settings. We separately compared the CADe with only highly confidence polyp labels of endoscopists, as well as total polyp labels by the endoscopists.

### Statistical analysis

All statistical analyses were performed in the Statistical Package for the Social Science (SPSS Inc., Chicago, IL, United States) software version 23.0. Continuous variables were presented as mean and standard deviation (SD), whereas categorical variables were reported as proportions and percentages. All the categorical variables were compared using the McNemar’s chi-square test. A two-sided *P*-value of 0.05 was the threshold for statistical significance.

## Results

### Baseline characteristics

Table 1 shows the patients and polyps characteristics. The study comprised 464 images from 116 patients with 116 polyps. The mean age of patients was 56.04 ± 10.41 years, and 64.66% (75/116) were male. The size of polyps ranged from 3 to 8 mm, mean size was 4.30 ± 1.50 mm. According to the Paris endoscopic classification of superficial neoplastic lesions (30), 76.73% (89/116) were type Is, 21.55% (25/116) were type Is-flat and 1.72% (2/116) were type Ip. 33.62% (39/119) polyps. 33.62% (39/116) of polyps were located in the ascending colon (including hepatic flexure), 25.86% (30/116) in the transverse colon, 18.97% (22/116) in the sigmoid colon, 12.93% (15/116) in the descending colon (including splenic flexure), and the remaining 8.62% (10/116) in the rectum.

**TABLE 1:** Baseline information.

Age, mean ( ± SD)	56.05 ± 10.83
Sex, n(%)	
Male	76 (65.52%)
Female	40 (34.48%)
Mean size (mm), mean ( ± SD)	4.50 ± 1.40
Paris classification, n(%)	
Is	86 (74.14%)
Is-flat	21 (18.10%)
Ip	2 (1.72%)
Isp	7 (6.04%)
Polyp location, n(%)	
Rectum	10 (8.62%)
Sigmoid colon	25 (21.55%)
Descending colon, including splenic flexure	16 (13.79%)
Transverse colon	29(25.00%)
Ascending colon, including hepatic flexure	36 (31.04%)
Polyp histology, n(%)	
Adenomatous*	81 (69.83%)
Hyperplastic	14 (12.07%)
Inflammatory	20 (17.24%)
Juvenile polyp	1 (0.86%)

*All adenomas were tubular adenoma with low grade dysplasia.

### Comparison of detection performance of CADe and endoscopists

#### Endsocopists vs. CADe: overall, fully inflated lumen and deflated lumen

**FIGURE 1:**
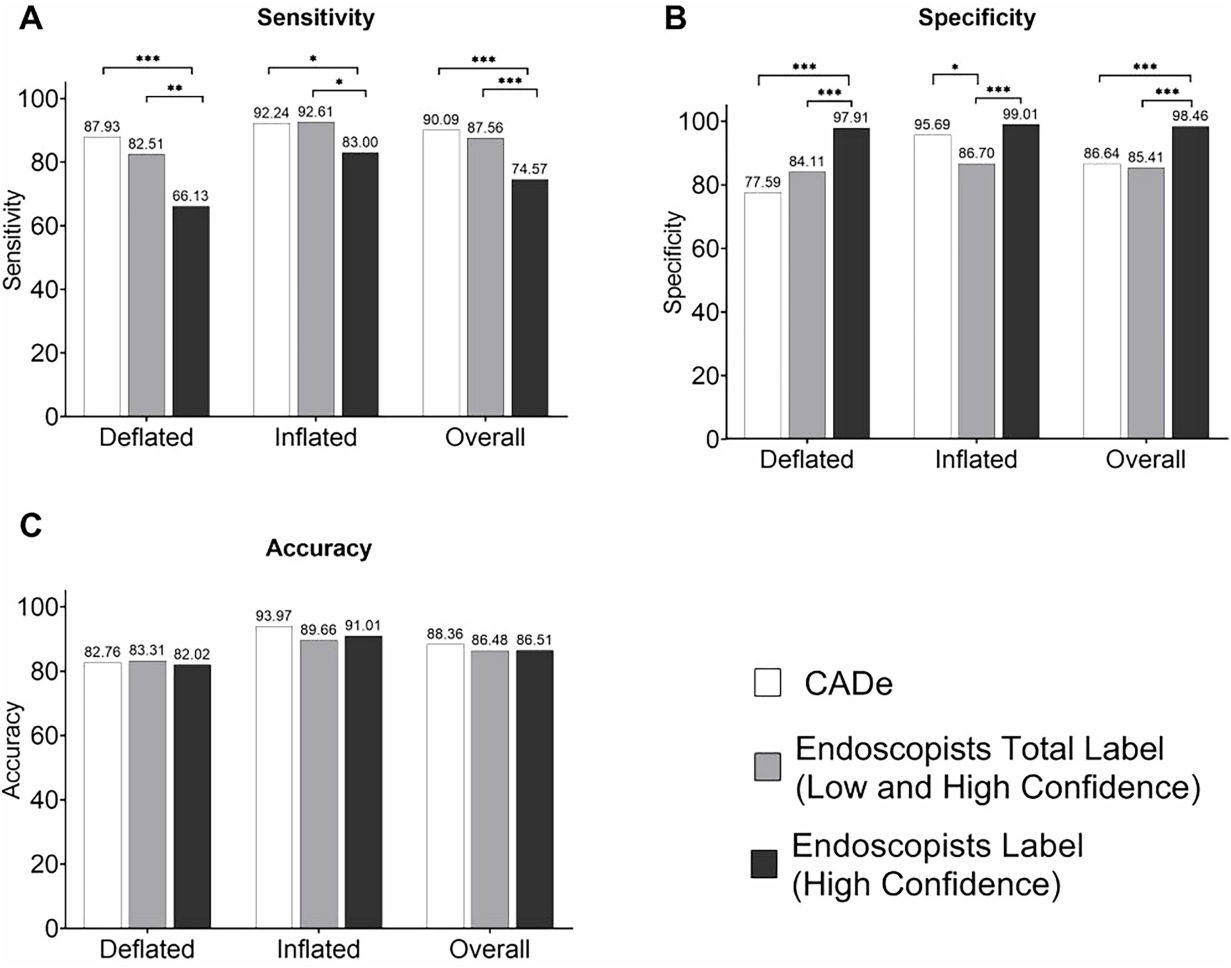
**(A)** Sensitivity: CADe (AI) vs. Endoscopists overall (Low and high confidence) vs. Endoscopists (only High confidence). **(B)** Specificity: CADe (AI) vs. Endoscopists overall (Low and high confidence) vs. Endoscopists (only High confidence). **(C)** Accuracy: CADe (AI) vs. Endoscopists overall (Low and high confidence) vs. Endoscopists (only High confidence). Figure legend significance note: *P < 0.05, **P < 0.01, ***P < 0.001. White bar: CADe system; Gray bar: Endoscopists total label (low and high confidence); Black bar: Endoscopists high-confidence label.

The CADe system and endoscopists exhibited sensitivity, specificity, and accuracy of 90.09% vs. 87.56% (*P* = 0.46), 86.64% vs. 85.41% (*P* = 0.79), and 88.36% vs. 86.48% (*P* = 0.43), respectively, across all labeled images (Figure 1 and Table 2).

**TABLE 2:** Diagnostic performance of computer-aided detection and endoscopists.

Condition	Outcome	CADe	Total (Low and high confidence)	*P*	CADe	High confidence	*P*
Overall	Sensitivity	90.09%	87.56%	0.4618	90.09%	74.57%	<0.0001***
	Specificity	86.64%	85.41%	0.7891	86.64%	98.46%	<0.0001***
	Accuracy	88.36%	86.48%	0.4289	88.36%	86.51%	0.4289
Inflated	Sensitivity	92.24%	92.61%	1.0000	92.24%	83.00%	0.0471*
	Specificity	95.69%	86.70%	0.0353*	95.69%	99.01%	0.2146
	Accuracy	93.97%	89.66%	0.1276	93.97%	91.01%	0.2915
Deflated	Sensitivity	87.93%	82.51%	0.3533	87.93%	66.13%	0.0002***
	Specificity	77.59%	84.11%	0.2411	77.59%	97.91%	<0.0001***
	Accuracy	82.76%	83.31%	1.0000	82.76%	82.02%	0.9031

*P < 0.05; ***P < 0.001.

In the test set with images taken in fully inflated lumen, sensitivity, specificity, and accuracy for the CADe system and endoscopists were 92.24% vs. 92.61% (*P* = 1.00), 95.69% vs. 86.70% (*P* = 0.04), and 93.97% vs. 89.66% (*P* = 0.13), respectively.

In the test set with images taken in deflated lumen, sensitivity, specificity, and accuracy for the CADe system and endoscopists were 87.93% vs. 82.51% (*P* = 0.35), 77.59% vs. 84.11% (*P* = 0.24), and 82.76% vs. 83.31% (*P* = 1.00).

**FIGURE 2:**
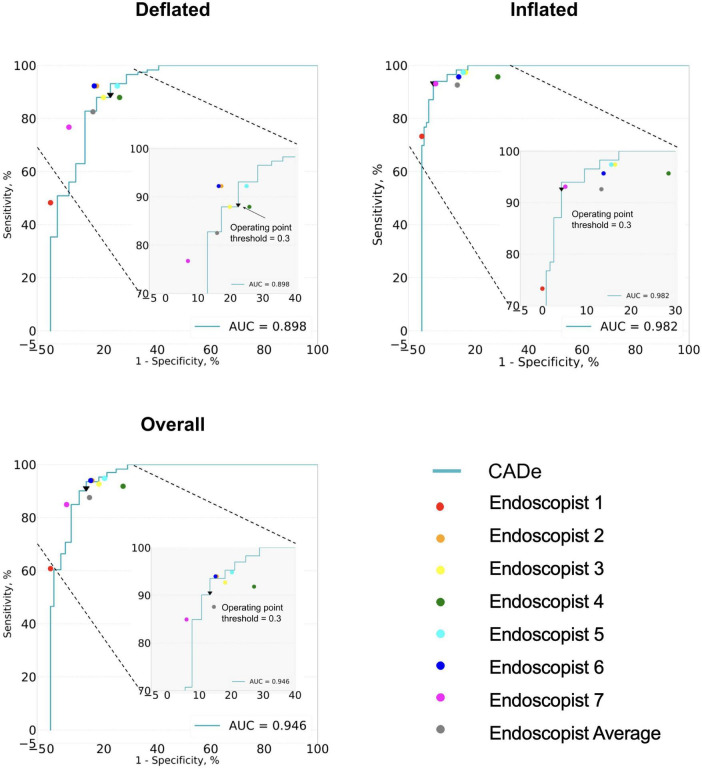
ROC of CADe (AI) vs. Endsocopists: Deflated colon, Inflated colon and Overall. ▼ refers to the operating point of 0.3.

The performance of each endoscopist in each subgroup could been seen in Figure 2 and Supplementary Table S1. The distribution of FP types for the CADe system and each endoscopist is shown in Supplementary Table S2; mucosal folds represented the predominant source of FPs for both CADe and endoscopists, although endoscopists also labeled a broader range of non-polyp structures (e.g., bubbles, reflections, liquid) as suspected polyps.

#### Endsocopists vs. CADe: endoscopists’ high confidence labeled polyps only

When analyzing only high confidence polyp lables by endoscopists, the endoscopists showed significantly higher specificity (98.46% vs. 86.64, *P* < 0.0001) and lower sensitivity (74.57% vs. 90.09%, *P* < 0.0001) than CADe system across all labeled images. There is no statistically significant difference in accuracy (86.51% vs. 88.36%, *P* = 0.43) between the two groups (Figure 1 and Table 2).

In the test set with images taken in fully inflated lumen, endoscopists’ high confidence labels had lower sensitivity (83.00% vs. 92.24%, *P* = 0.047) than CADe system, the specificity (99.01% vs. 95.69%, *P* = 0.21) and accuracy (91.01% vs. 93.97%, *P* = 0.29) for endoscopists were consistent with those of the CADe system.

In the test set with images taken in deflated lumen, endoscopists’ high confidence labels had significantly lower sensitivity (66.13% vs. 87.93%, *P* = 0.0002) and specificity (97.91% vs. 77.59%, *P* < 0.0001) than CADe system, the accuracy showed no difference between the two groups (82.02% vs. 82.76%, *P* = 0.90).

## Discussion

Both experts consensus and surveys targeting endoscopists have shown that FPs are currently an important issue affecting the adoption and clinical use of CADe colon polyp detection algorithms (31, 32). To our knowledge, this is the first study directly comparing CADe FPs with endoscopist FPs.

In this study, we found that across all scenarios examined (both optimal/inflated lumen images and sub-optimal/underinflated lumen images), there was similar diagnostic sensitivity, specificity and accuracy between CADe systems and experienced endoscopists. This is also in line with the results of a clinical multicenter RCT that we previously published, in which we found that a trainee “second observer” had a higher polyp FPs rate and different FP types compared to CADe in a real-world clinical setting (29).

Secondly, in optimal conditions characterized by complete intestinal inflation, both CADe system and endoscopists demonstrate very high polyp detection sensitivity. However, CADe exhibits significantly greater specificity. These findings suggest that in colonoscopy optimal conditions, CADe may actually yield fewer FPs than endoscopists themselves, while maintaining high polyp detection sensitivity. In more challenging colonoscopy conditions characterized by underinflated lumen wherein the colon wall may be thickened or partially wrinkled, making polyps more difficult to distinguish, the overall diagnostic accuracy is comparable between CADe and endoscopists. In challenging conditions, the visual features of polyps are reduced, resulting in a decrease in sensitivity for both endoscopists and CADe. However, considering that human endoscopists may have diagnostic bias or a mentality of avoiding difficulties (33–35), the true sensitivity of endoscopists may be lower in more challenging real clinical setting.

Next, by separately analyzing the polyps that endoscopists were highly certain of, we can see the enhancement in specificity is counterbalanced by a more notable decrease in sensitivity. The ultimate goal of colonoscopy screening is polyp detection, making lesion detection rate and diagnostic sensitivity the most fundamental endpoints that endoscopists must prioritize above all else. Thus, strategies that achieve a low FPR at the cost of an increased rate of missed polyps are clinically inefficient and therefore unacceptable. These findings underscore the critical importance of maintaining an appropriate threshold of reasonable suspicion during colonoscopic polyp detection, and highlight the core clinical principle of “better to suspect than to miss” adopted by meticulous endoscopists with higher detection rate in routine practice.

**FIGURE 3:**
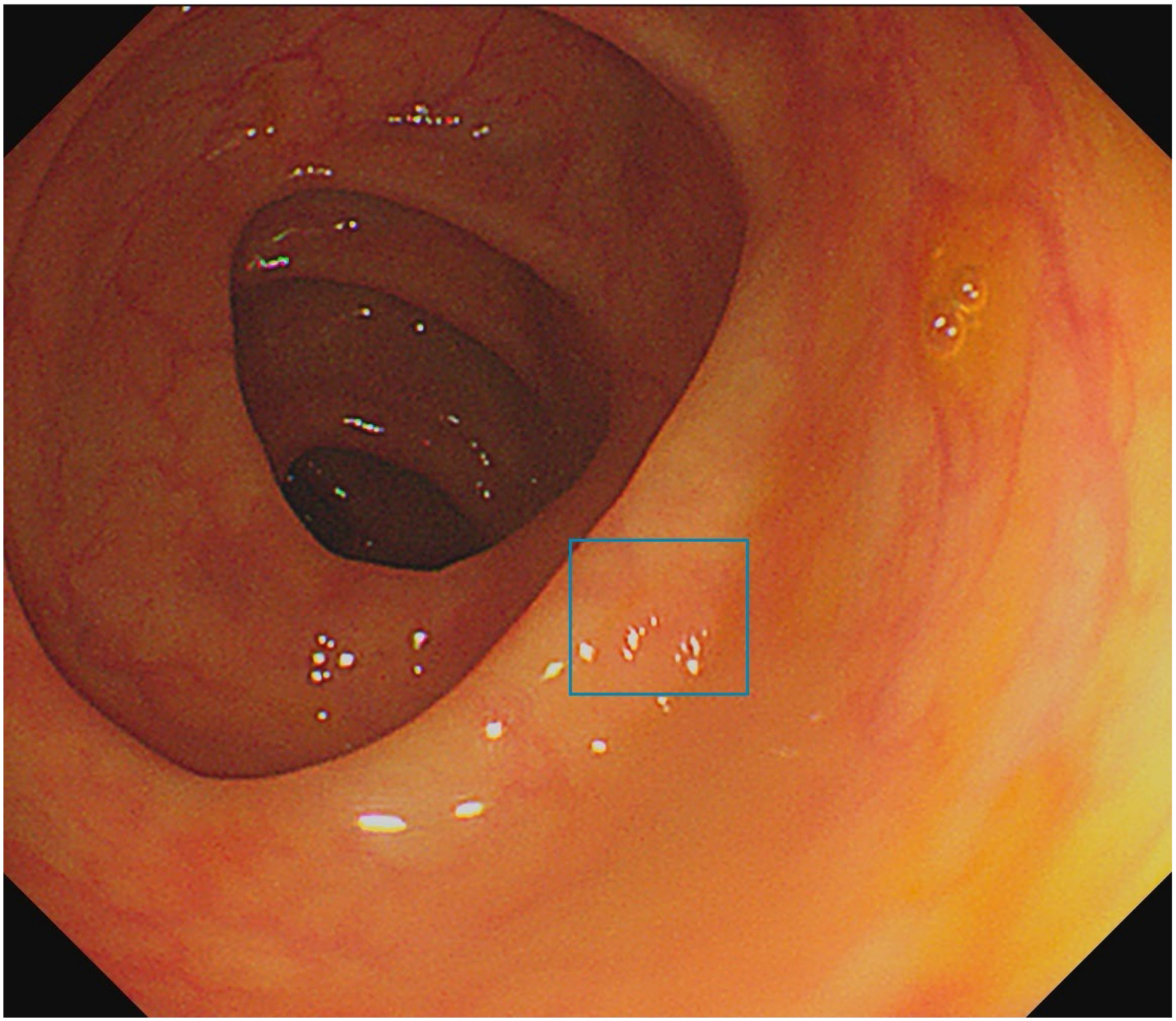
Non close distance image, a meaningful alert: Subtle color difference and a slightly elevated feeling. The final close-up observation demonstrated a polyp, which was confirmed by pathological examination to be a tubular adenoma. If things in the box is ultimately proven not a polyp, this alert cannot be considered meaningless FPs.

**FIGURE 4:**
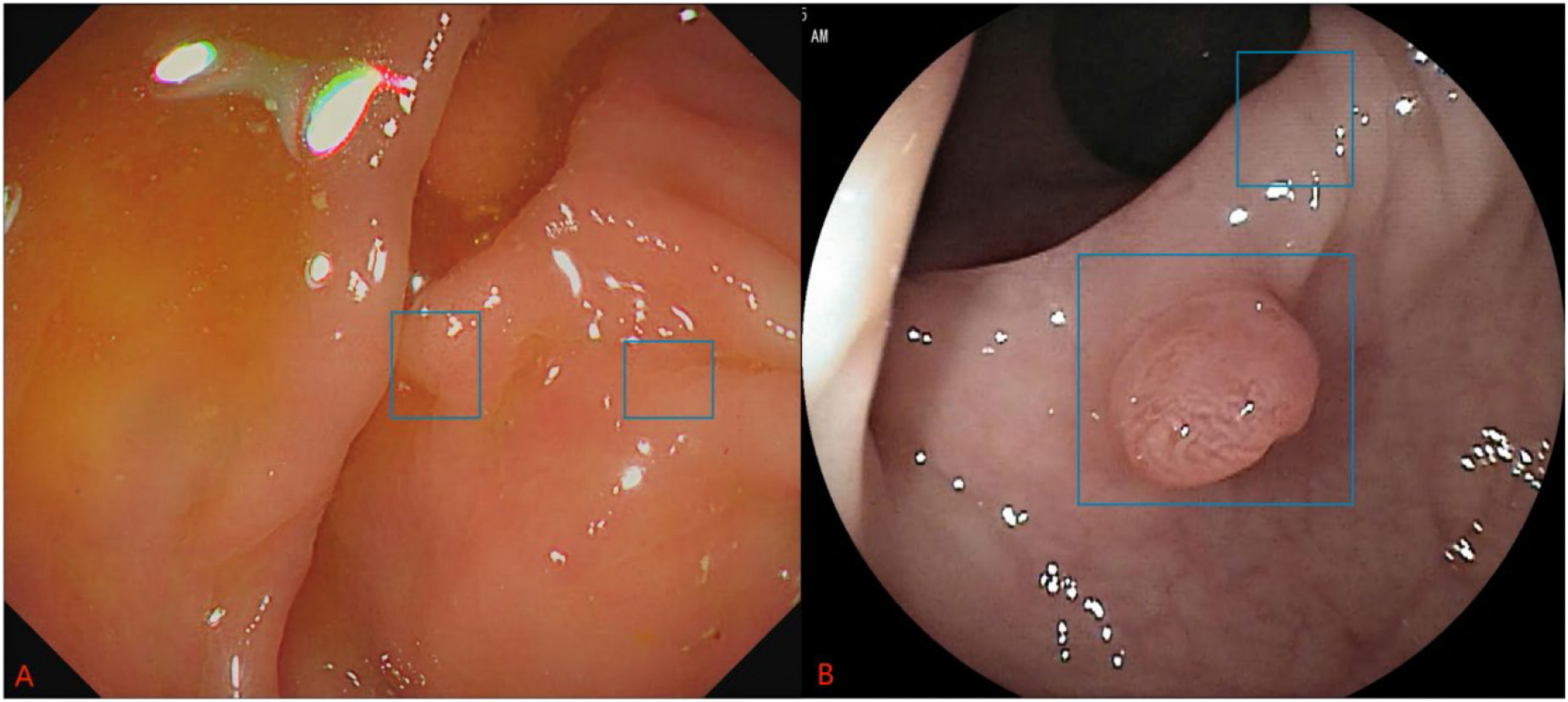
**(A)** The right box shows a very obvious FP. **(B)** The upper right box is a very obvious FP.

When employing a CADe system, endoscopists harboring skepticism toward its efficacy may sometimes thoughtlessly dismiss certain alert boxes as FPs, failing to meticulously scrutinize them at close range, however, not each disregarded alert box should be invariably represent true FPs. For CADe systems in gastrointestinal endoscopy, a non-negligible subset of generated alerts presents visual features that are indistinguishable with limited pixel resolution, including polypoid protrusive morphology, mucosal erythema, and circumscribed margin. Even if these alerts are confirmed as FPs after close-range evaluation during colonoscopy, they still warrant deliberate close-up examination at the time of detection—most notably when the alerted region is positioned distally from the endoscope camera (Figure 3). This subset of FPs therefore retains non-trivial clinical value, in direct contrast to truly non-informative FPs, which are defined as immediately recognizable “single-glance errors” captured under close-range visualization during colonoscopy (Figure 4). Accordingly, a critical unmet need in future CADe research is to avoid the conceptual conflation of clinically meaningful FPs (mFPs) and non-meaningful FPs (nFPs), with dedicated efforts to systematically re-evaluate the clinical and diagnostic value of mFPs.

This study reveals high-level CADe systems may not necessarily have more FPs than endoscopists. Additionally, we seek to further discuss the cognitive variances inherent in endoscopists’ interpretation of human-generated FPs versus those generated by AI and possible ways to mitigate the interference of CADe alert boxes on endoscopists.

With the increasingly widespread clinical adoption of CADe systems, a considerable number of endoscopists hold the view that CADe generates an excessive number of FPs, which introduce unnecessary distraction and operational frustration. However, meticulous endoscopists never regard suspicious regions that they have ultimately ruled out as a human-analogous “FP,” nor do they perceive such regions as a source of irritation or disruption. Beyond larger polyps that can be definitively identified at a single glance under close-range visualization, endoscopists typically follow a two-step diagnostic workflow for all other potential polypoid lesions. First, they identify visual features with polypoid morphology that cannot be immediately characterized, triggering suspicion of a polyp. Second, they maneuver the colonoscope to acquire close-range views of the suspicious region, to ultimately confirm or rule out the presence of a polyp (29). These two distinct diagnostic paradigms—“immediate single-glance confirmation” and “suspicion followed by systematic confirmation or exclusion”—are associated with fundamentally different cognitive and psychological processes in endoscopists, consistent with the dual-process theory of clinical reasoning (intuitive System versus analytical System) (36–38). As such, endoscopists experience no psychological burden from their own intrinsic suspicion-raising workflow. However, the alert boxes generated by CADe systems do not incorporate this hierarchical diagnostic logic: CADe delivers visually and functionally identical alert boxes for both definitively identifiable polyps and indeterminate, suspicious findings. Consequently, the complete parity between FP and TP alerts creates a cognitive bias among endoscopists, leading to the subjective perception of an excessively high FPR (39). Therefore, on the premise of ensuring that CADe systems maintain a TP detection rate no lower than that of human endoscopists, while preserving the alerting level for clinically meaningful suspicious findings, such systems must adopt an interaction logic aligned with clinicians’ native diagnostic thinking processes. Specifically, CADe should differentiate between definitively identifiable polyps and indeterminate suspicious findings via variations in the color, shape, and other visual attributes of alert boxes, to facilitate more informed clinical decision-making during endoscopic procedures.

This study is subject to several limitations. Firstly, the sample size is relatively small, potentially limiting its representativeness of the diversity of polyp morphologies, locations, and colon lumen environments encountered in real-world settings. Secondly, being a single-center study with a limited number of participating endoscopists, it may not fully capture the diversity of endoscopist styles and expertise levels worldwide. Thirdly, the evaluation was restricted to a single CADe system, and thus, the generalizability of the findings to other mainstream high-performance CADe systems may be limited. Fourthly, while this study shows that a high-performance CADe system does not produce more FPs than endoscopists, still image-derived findings have limited generalizability to real-world dynamic video scenarios. However, due to the subjectivity in defining FPs for endoscopists and CADe in studies under real clinical conditions, still image based research still has advantages in controlling variables and reducing rule-induced bias. In addition, our multicenter clinical study (29) has preliminarily found that CADe generates fewer FPs than endoscopists in real-world settings, though this conclusion requires further data validation. Lastly, it should be noted that although this study found that CADe did not have more FPs than endoscopists, the inclusion of challenging images such as underinflated lumen images, posed greater challenges to CADe, which may be the reason that CADe merely matched, and the overall detection accuracy and sensitivity of CADe did not significantly exceed, that of experienced endoscopists. Improving CADe performance in sub-optimal colon environments is an important research topic in the future.

In conclusion, this study found that a state-of-the-art CADe system for colon polyp detection has a similar FPR to experienced human endoscopists. Future research on CADe FPs should focus not only on reducing the number of CADe FPs, but on developing a more nuanced understanding of the role of FPs on performance of the human-AI hybrid, paving the road for innovations in CADe user interfaces.

## Data Availability

The original contributions presented in this study are included in this article/Supplementary material, further inquiries can be directed to the corresponding author.
