# Correction: Advanced computer-aided detection system exhibits no more false positives than experienced endoscopists in an image-based comparative study of colon polyps

**DOI:** 10.3389/fmed.2026.1924923

**Published:** 2026-07-09

**Authors:** Frontiers Production Office

**Affiliations:** Frontiers Media SA, Lausanne, Switzerland

**Keywords:** artificial intelligence, colon polyp, colonoscopy, computer aided detection, false positive

There was a mistake in Figures 3, 4 as published. The image for Figure 3 is the one for Figure 4 and *vice versa*. The corrected figures appears below.

**Figure 3:**
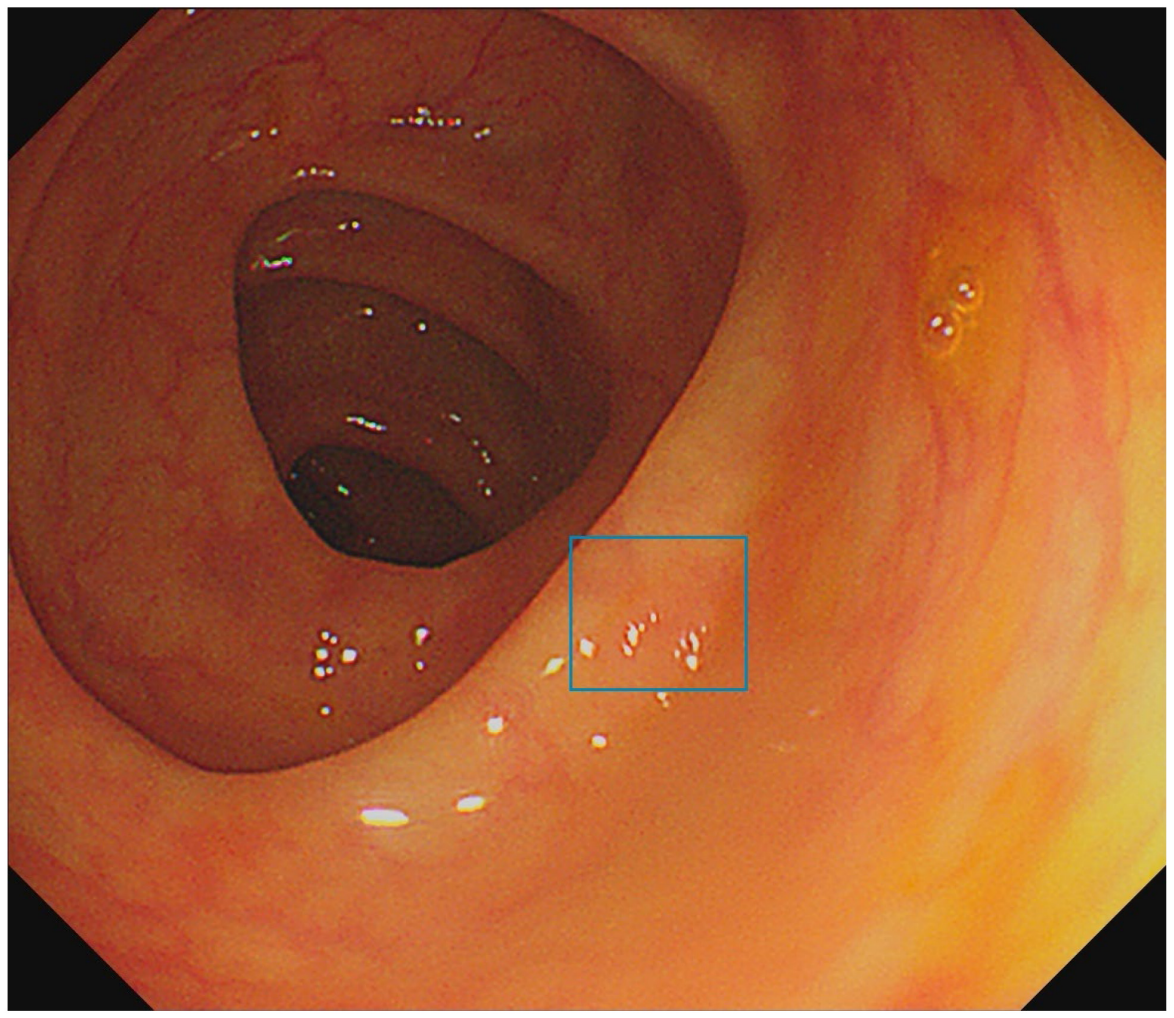
Non close distance image, a meaningful alert: subtle color difference and a slightly elevated feeling. The final close-up observation demonstrated a polyp, which was confirmed by pathological examination to be a tubular adenoma. If things in the box is ultimately proven not a polyp, this alert cannot be considered meaningless FPs.

**Figure 4:**
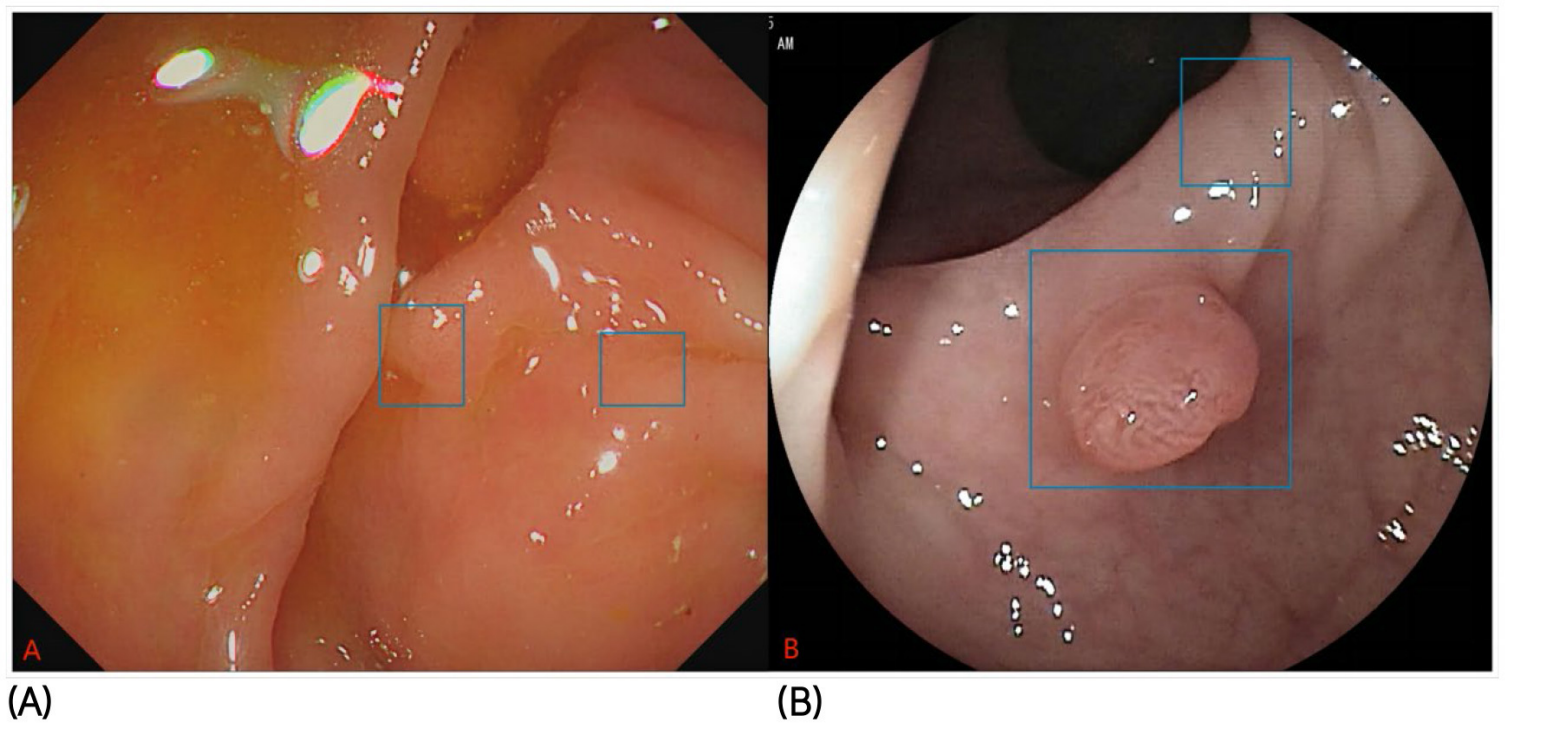
**(A)** The right box shows a very obvious FP. **(B)** The upper right box is a very obvious FP.

The original version of this article has been updated.

## Generative AI statement

Any alternative text (alt text) provided alongside figures in this article has been generated by Frontiers with the support of artificial intelligence and reasonable efforts have been made to ensure accuracy, including review by the authors wherever possible. If you identify any issues, please contact us.

