# Supplementary material for: Advanced computer-aided detection system exhibits no more false positives than experienced endoscopists in an image-based comparative study of colon polyps
Source: Front Med (Lausanne). 2026 Jun 11;13:1793448. doi: 10.3389/fmed.2026.1793448 (PMC13293802; doi:10.3389/fmed.2026.1793448)
Supplement: Supplementary file 1 [file Table_1.docx]

| **Total**  **(Low and high confidence)** | **Performance Metric** | **1** | **2** | **3** | **4** | **5** | **6** | **7** | **Average** |
| --- | --- | --- | --- | --- | --- | --- | --- | --- | --- |
| Overall | sensitivity | 60.78% | 93.97% | 92.67% | 91.81% | 94.83% | 93.97% | 84.91% | 87.56% |
|  | specificity | 100.00% | 84.48% | 81.90% | 72.84% | 79.74% | 84.91% | 93.97% | 85.41% |
|  | accuracy | 80.39% | 89.22% | 87.28% | 82.33% | 87.28% | 89.44% | 89.44% | 86.48% |
| inflated | sensitivity | 73.28% | 95.69% | 97.41% | 95.69% | 97.41% | 95.69% | 93.10% | 92.61% |
|  | specificity | 100.00% | 86.21% | 83.62% | 71.55% | 84.48% | 86.21% | 94.83% | 86.70% |
|  | accuracy | 86.64% | 90.95% | 90.52% | 83.62% | 90.95% | 90.95% | 93.97% | 89.66% |
| deflated | sensitivity | 48.28% | 92.24% | 87.93% | 87.93% | 92.24% | 92.24% | 76.72% | 82.51% |
|  | specificity | 100.00% | 82.76% | 80.17% | 74.14% | 75.00% | 83.62% | 93.10% | 84.11% |
|  | accuracy | 74.14% | 87.50% | 84.05% | 81.03% | 83.62% | 87.93% | 84.91% | 83.31% |
|  |  |  |  |  |  |  |  |  |  |
|  |  |  |  |  |  |  |  |  |  |
| **High confidence** | **Performance Metric** | **1** | **2** | **3** | **4** | **5** | **6** | **7** | **Average** |
| Overall | sensitivity | 45.69% | 84.91% | 68.53% | 77.59% | 81.47% | 84.91% | 78.88% | 74.57% |
|  | specificity | 100.00% | 96.55% | 100.00% | 98.71% | 98.28% | 96.55% | 99.14% | 98.46% |
|  | accuracy | 72.84% | 90.73% | 84.27% | 88.15% | 89.87% | 90.73% | 89.01% | 86.51% |
| inflated | sensitivity | 55.17% | 90.52% | 81.03% | 89.66% | 87.07% | 90.52% | 87.07% | 83.00% |
|  | specificity | 100.00% | 98.28% | 100.00% | 99.14% | 99.14% | 98.28% | 98.28% | 99.01% |
|  | accuracy | 77.59% | 94.40% | 90.52% | 94.40% | 93.10% | 94.40% | 92.67% | 91.01% |
| deflated | sensitivity | 36.21% | 79.31% | 56.03% | 65.52% | 75.86% | 79.31% | 70.69% | 66.13% |
|  | specificity | 100.00% | 94.83% | 100.00% | 98.28% | 97.41% | 94.83% | 100.00% | 97.91% |
|  | accuracy | 68.10% | 87.07% | 78.02% | 81.90% | 86.64% | 87.07% | 85.34% | 82.02% |

**Supplementary Table 1**. Detection performance of the 7 endoscopists participating in the study.
