# Supplementary material for: Advanced computer-aided detection system exhibits no more false positives than experienced endoscopists in an image-based comparative study of colon polyps
Source: Front Med (Lausanne). 2026 Jun 11;13:1793448. doi: 10.3389/fmed.2026.1793448 (PMC13293802; doi:10.3389/fmed.2026.1793448)
Supplement: Supplementary file 2 [file Table_2.docx]

| **Cause of False positives (FPs), n (%)** | **CADe** | **Endoscopist 1** | **Endoscopist 2** | **Endoscopist 3** | **Endoscopist 4** | **Endoscopist 5** | **Endoscopist 6** | **Endoscopist 7** |
| --- | --- | --- | --- | --- | --- | --- | --- | --- |
| **Total FPs** | 42 (100%) | 2(100%) | 47(100%) | 56(100%) | 86(100%) | 64(100%) | 46(100%) | 21(100%) |
| Mucosal Folds | 38 (90.48%) | 0 | 38 (80.85%) | 41 (73.21%) | 63 (73.26%) | 56 (87.5%) | 41 (89.13%) | 15 (71.43%) |
| Normal mucosa | 1 (2.38%) | 0 | 1 (2.13%) | 3 (5.36%) | 9 (10.47%) | 4 (6.25%) | 3 (6.52%) | 5 (23.81%) |
| Ileocecal valve | 0 | 0 | 0 | 0 | 1 (1.16%) | 1 (1.56%) | 0 | 0 |
| Abrasion | 1 (2.38) | 0 | 1 (2.13%) | 0 | 1 (1.16%) | 0 | 0 | 0 |
| Bubbles | 1 (2.38%) | 1 (50%) | 2 (4.25%) | 2 (3.57%) | 2 (2.33%) | 1 (1.56%) | 0 | 1 (4.76%) |
| Fecal | 0 | 0 | 1 (2.13%) | 1 (1.79%) | 0 | 0 | 0 | 0 |
| Mucus | 0 | 0 | 0 | 1 (1.79%) | 1 (1.16%) | 0 | 0 | 0 |
| Liquid | 1 (2.38%) | 0 | 0 | 2 (3.57%) | 4 (4.65%) | 0 | 0 | 0 |
| Reflection | 0 | 1 (50%) | 2 (4.25%) | 5 (8.92%) | 3 (3.49%) | 0 | 1 (2.17%) | 0 |
| Suction artifact | 0 | 0 | 1 (2.13%) | 1 (1.79%) | 1 (1.16%) | 1 (1.56%) | 0 | 0 |
| Local inflammation | 0 | 0 | 1 (2.13%) | 0 | 1 (1.16%) | 1 (1.56%) | 1 (2.17%) | 0 |

**Supplementary Material Table S2. Characteristics of false positives by CADe system and endoscopists**
